# Anti-Angiogenic and Anti-Inflammatory Effects of SERPINA3K on Corneal Injury

**DOI:** 10.1371/journal.pone.0016712

**Published:** 2011-01-31

**Authors:** Xiaochen Liu, Zhirong Lin, Tong Zhou, Ronrong Zong, Hui He, Zhen Liu, Jian-xing Ma, Zuguo Liu, Yueping Zhou

**Affiliations:** 1 Eye Institute of Xiamen University, Fujian Provincial Key Laboratory of Ophthalmology and Visual Science, Xiamen, China; 2 Department of Physiology, The University of Oklahoma Health Sciences Center, Oklahoma City, Oklahoma, United States of America; Istituto Dermopatico dell'Immacolata, Italy

## Abstract

SERPINA3K is a member of the serine proteinase inhibitor (SERPIN) family. Here we evaluated the therapeutic effects of SERPINA3K on neovascularization and inflammation in a rat cornea alkali burn model that is commonly employed to study corneal wounding. Topical treatment of the injured rat cornea with SERPINA3K (20 µg/eye/day) for 7 days significantly decreased the neovascular area, compared with the groups treated with BSA or PBS. The SERPINA3K treatment also ameliorated the corneal inflammation as evaluated by the inflammatory index. Furthermore, SERPINA3K enhanced the recovery of corneal epithelium after the alkali injury. Toward the mechanism of action, SERPINA3K down-regulated the expression of the pro-angiogenic and pro-inflammatory factors, vascular endothelial growth factor and tumor necrosis factor-α and up-regulated the expression of the anti-angiogenic factor, pigment epithelium-derived factor. SERPINA3K specifically inhibited growth of vascular endothelial cells. Meanwhile, SERPINA3K significantly up-regulated the expression of EGFR in the corneal epithelium. These findings suggest that SERPINA3K has therapeutic potential for corneal inflammation and NV.

## Introduction

Corneal injury is a common ophthalmologic disease and lacks satisfactory therapies [1]. Angiogenesis or neovascularization (NV) and inflammation are important pathologic events during the corneal wounding and healing [2], [3]. Vascular endothelial growth factor (VEGF) and tumor necrosis factor-α (TNF-α) are known to play important roles in the pathogenesis of NV and inflammations [4]–[6].

It has been shown that there are counter-balance systems which regulate angiogenesis or NV, the pro-angiogenic factors, such as VEGF, and the anti-angiogenic factors, such as pigment epithelium-derived factor (PEDF) [7]–[10]. A major category of the natural angiogenic inhibitor is the serine proteinase inhibitor (SERPIN) family, as a number of serpin members (e.g. PEDF, SERPINA3K, maspin, α1-antitrypsin, anti-thrombin) have been identified as endogenous angiogenic inhibitors [11]–[15].

SERPINA3K, also known as kallikrein-binding protein (KBP), belongs to the SERPIN family. It is expressed in the liver, kidney and ocular tissues, etc [16]–[18]. SERPINA3K was first identified as a specific inhibitor of tissue kallikrein. It specifically binds with tissue kallikrein to form a covalent complex and inhibit its proteolytic activities. Later studies reported that it has other functions independent of inhibition of tissue kallikrein [19], [20]. SERPINA3K has effects of anti-angiogenesis, anti-inflammation, anti-fibrosis and anti-oxidative stress in the retina [21], [22]. It was previously reported that SERPINA3K blocks the binding of VEGF to VEGF receptor through competing on heparin binding, since both VEGF and SERPINA3K are heparin-binding proteins [20]. Lately, it was suggested that SERPINA3K blocks the canonical Wnt pathway, and consequently, down-regulates expression of pro- inflammatory and pro-angiogenic factors such as VEGF, TNF-α and ICAM-1, which may represent a unifying mechanism underlying its anti-angiogenesis and anti-inflammation effects [23]. However, little is known about potential application of SERPINA3K in the corneal injury.

In this present study, we for the first time investigated the anti-angiogenic and anti-inflammatory effects of SERPINA3K in the cornea using an alkali burn model and explored its mechanism of action.

## Materials and Methods

Materials: The antibodies of VEGF, PEDF, TNF-α, epidermal growth factor receptor (EGFR) were purchased from Abcam, Cambridge, MA, USA. Antibody for β-actin was purchased from Bio-Rad, Hercules, CA, USA. AlexaFluor488-conjugated IgG was purchased from Invitrogen, Carlsbad, CA, USA. MTT [3-(4,5-dimethylthiazol-2-yl)-2,5- diphenyltetrazolium bromide] was purchased from Sigma, Saint Louis, MO, USA. Fluorescein sodium solution was purchased from Jingming Co. Ltd, Tianjing, China).

### Purification of SERPINA3K

The SERPINA3K/pET28 plasmid expressing SERPINA3K was introduced into *E. coli* strain BL21. The vector provides a signal peptide that enables the recombinant protein to enter the periplasmic space. The expression and purification followed the protocol recommended by G.E Company with some modifications. Briefly, expression was induced by the addition of isoprupylthio-β-galactoside (IPTG) and carried out overnight at 25°C. Periplasmic proteins were released by digestion with lysozyme and separated from cells by centrifugation. SERPINA3K was purified by passing through the His-Bind column (G.E). The purity of recombinant SERPINA3K was examined by SDS-PAGE. Endotoxin concentrations were monitored using a limulus amebocyte kit. Activity of the purified protein was examined by MTT assay using primary Human Umbilical Vein Endothelial Cells (HUVEC).

### Procedure of corneal alkali burn

Twenty-four male Wistar rats (150–180 g) were purchased from Shanghai Shilaike Laboratory Animal Co, Ltd., Shanghai, China. Animal experiments were carefully conducted in accordance with the ARVO Statement for the Use of Animals in Ophthalmic and Vision Research and the animal experimental procedures were approved by the Experimental Animal Committee of Xiamen University (approval ID: XMUMC2009-02-1). Animals were housed in a temperature, humidity, and light controlled room. Food and water were available ad libitum. The procedures of corneal alkali burn were briefly as follows: The rats were anesthetized with an intraperitoneal injection of 40 mg/kg pentobarbital and received topically administration with a drop of tetracaine. A round filter paper (3.5 mm in diameter) soaked with 1 N NaOH or PBS was placed on the center of the corneal surface for 30 sec to induce alkali burn. The ocular surface was then rinsed with 10 ml PBS. Rats were randomly divided into 4 groups (n = 6). Group 1: Control group, without alkali burn; Group 2: alkali burn with PBS treatment (10 µl, 4 times per day); Group 3: alkali burn with BSA treatment (500 µg/ml in volume of 10 µl, 4 times per day) and group 4: alkali burn with SERPINA3K treatment (500 µg/ml in volume of 10 µl, 4 times per day, daily, in a dose of 20 µg/eye/day). All eyes were observed on day 1, 2, 5 and 8 with slit lamp microscopy as described below for evaluation of corneal NV and inflammation and measurement of corneal epithelium damage. The rats were sacrificed on postoperative day 8, eye balls were removed, and the corneas were dissected and stored at −80°C for histological and immunological studies.

For the histology examination and immunofluorescent staining, the corneas were embedded in OCT, cross-sectioned, Hematoxylin and Eosin staining (H&E staining) was conducted or the immunofluorescent staining was performed as described below.

For the Western blot analysis, the whole cornea tissue including limbal (about 0.5 mm width) area was carefully dissected and Western blotting was conducted as described below.

### Evaluation of NV and inflammation

Corneal NV (NV) was quantified as previously described with modifications [24]. Briefly, all eyes were examined by a blinded ophthalmologist under slit lamp microscope on day 1, 2, 5 and 8 after alkali burn. The corneal image was divided into 4 quarters. The vessel length of each quarter (L*_i_*, *i* = 1–4) was measured using a vernier caliper. The corneal NV area (A) was calculated using the following equation: A = Σ_i = 1–4_ 3.1416×{R^2^−(R−L_i_)^2^}. (R is the radius of rat cornea. R = 3.5mm, taken from the measurements of 20 rat corneas).

Inflammatory response was evaluated by slit lamp. Serial photographs of the cornea were taken. The inflammatory index was measured as previously described [25]. Briefly, the inflammatory index was analyzed, based on the following parameters: ciliary hyperemia (absent, 0; present but less than 1 mm, 1; present between 1 and 2 mm, 2; present and more than 2 mm, 3); central corneal edema (absent, 0; present with visible iris details, 1; present without visible iris details, 2; present without visible pupil, 3); and peripheral corneal edema (absent, 0; present with visible iris details, 1; present without visible iris details, 2; present with no visible iris, 3). The final inflammatory index result was obtained by summing the crosses of the different parameters divided by a factor of 9 [25].

### Measurement of corneal epithelium damage

On days 1, 2, 5 and 8 after the alkali burn, all eyes were examined by a masked ophthalmologist. The fluorescein tests were performed as follows [26]: 5 µl 0.1% fluorescein sodium solution was instilled in each eye of the rats. After 60s, the eyes were examined with cobalt blue light. Pictures were taken with a digital camera. The extent of corneal damage was scored according to the following scale: 0, no staining; 0.5, slight punctate staining; 1, diffuse punctate staining; 2, diffuse staining covering less than one third of the cornea; 3, diffuse staining covering more than one-third of the cornea; and 4, staining covering more than two-third of the cornea.

### Immunofluorescent staining

Twelve cryostat sections (6 µm in thickness) of each cornea were fixed in acetone, blocked, and incubated with an anti-EGFR antibody (1∶400) at 4°C overnight. After further incubation in AlexaFluor488-conjugated IgG (1∶1000), sections were counterstained with DAPI, mounted, and photographed using a confocal laser scanning microscope (Fluoview 1000, Olympus, Tokyo, Japan). Negative controls without primary antibodies were performed.

### Western blot analysis

Proteins of cornea from each group were extracted with cold PIPA buffer. Equal amounts of proteins extracted from lysates were subjected to electrophoresis on 8% Tricine gels and then electrophorectially transferred to PVDF membranes. After 1h blocking in 5% BSA, the blots were incubated with primary antibodies to VEGF (1∶500), PEDF (1∶200), TNF-α (1∶700), EGFR (1∶5000), β-actin (1∶5000) as a loading control. After three times washes with Tris-buffered saline with 0.05% Tween 20 for 10 min each, the membranes were incubated with HPR conjugated goat anti-rabbit and IgG (1∶10000) for 1h at room temperature. The specific binds were visualized by enhanced chemiluminescence reagents and recorded on film.

### Cell culture

Human Corneal Epithelial (HCE) cells were obtained from RIKEN Biosource Center (Tokyo, Japan) and were cultured in supplemented hormonal epithelial medium (SHEM), which comprises DMEM-F12 supplemented with 15% heat-inactivated fetal bovine serum, bovine insulin (5 µg/ml), recombinant human EGF (10 ng/ml) and 1% penicillin and streptomycin. For cell viability assay, the HCE cells were plated at a density of 1×10^4^ cells per well in 96-well culture plates. When the HCE cells were cultured to 70% confluency, the medium was removed and changed in the medium in the present of SERPINA3K at concentration of 20, 40, 80, 160, 320 nM and the medium in the absent of SERPINA3K. The MTT assay was performed after 72 hrs.

Primary HUVEC were purchase from Cascade Biologics Company (USA). They were cultured in medium M-200 supplemented with Low Serum Growth Supplement (LSGS), 1% penicillin and streptomycin. The seeding density was between 2.5×10^3^ cells/cm^2^ and 1×10^5^ cells/cm^2^. The media were changed every 3 days. When the HUVEC were cultured to 70% confluency, the medium was removed and changed in the medium containing SERPINA3K at concentration of 20, 40, 80, 160, 320 nM and the medium containing the same amount of BSA. The MTT assay was performed after 72 hrs.

### MTT assay

Briefly, 100 µl of 1 mg/ml MTT [3-(4,5-dimethylthiazol-2-yl)-2,5- diphenyltetrazolium bromide] constituted in culture media was added into each well, followed by incubation for 4h at 37°C in the dark. After incubation, the MTT solution was removed and the stained cells were washed twice in PBS followed by air-drying. The MTT-formazan products were extracted with 100 µl of DMSO in the dark at room temperature. The absorbance was measured sepctrophotometrically at 570 nm using a Bio Tek ELX800 microplate reader (USA).

### Statistical analysis

The statistical analysis of NV, inflammation and epithelium damage was performed with two-way analysis of variance test (ANOVA), followed by the Bonferroni post hoc analysis to compare the differences between the groups. The statistical analysis of all Western blotting results was conducted with one-way analysis of ANOVA, followed by post hoc analysis Tukey test to compare the differences between the groups. The data of MTT were analyzed with student t-test. P<0.05 was considered statistically significant.

## Results

### Effects of SERPINA3K on corneal NV

**Figure 1:**
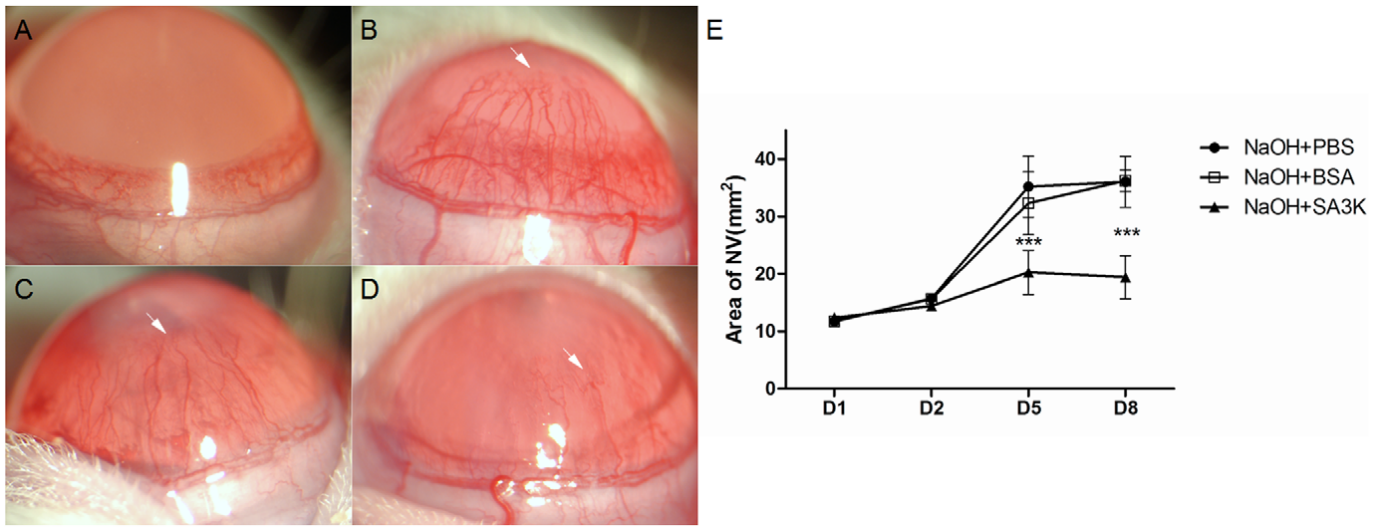
Effects of SERPINA3K on corneal NV (NV). (**A–D**) Representative images of NV on day 8 after alkali burn. (**A**) control group, without alkali burn, (**B**) alkali burn with PBS treatment; (**C**) alkali burn with BSA treatment; (**D**) alkali burn with SERPINA3K (SA3K) treatment (20 µg/eye/day). The new vessels were seen in the corneas (**B–D**) and the new vessels were reduced in (**D**), compared to (**B**) and (**C**), as the arrow indicates. (**E**) The statistic comparison of NV between the groups (data are presented as Mean±SEM, n = 6 in each group). The area of NV was significantly reduced in the SERPINA3K treated group on day 5 and 8, compared to PBS or BSA treated groups. (*** p<0.001).

On day 8 after alkali burn, significant NV was observed in the cornea treated with the PBS or BSA. The corneas treated with SERPINA3K showed apparently less NV (Fig. 1D), compared with the PBS or BSA-treated groups (Fig. 1B, 1C). The NV was quantified using measurement of area of NV, which showed that the NV area in the SERPINA3K-treated group was significantly reduced on both days 5 and 8, compared with the PBS or BSA-treated groups (Fig. 1E, data are presented as Mean±SEM, n = 6 in each group, *** p<0.001 ), suggesting an inhibitory effect on the corneal NV.

### Anti-inflammatory effects of SERPINA3K in the cornea

**Figure 2:**
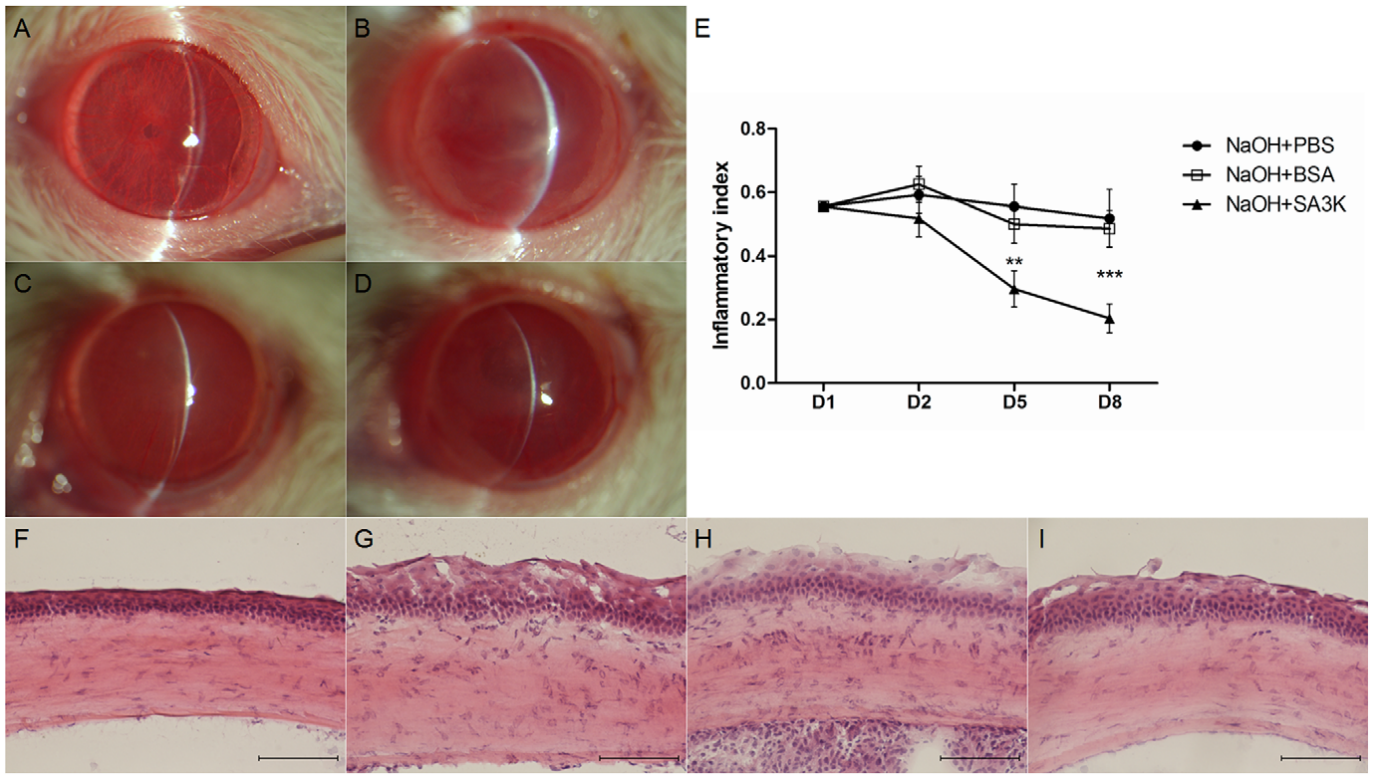
Anti-inflammatory effects of SERPINA3K in cornea. (**A–D**) Representative slit lamp images of the cornea on day 8 after burn. (**A**) control group, without alkali burn (**B**) alkali burn with PBS treatment; (**C**) alkali burn with BSA treatment; (**D**) alkali burn with SERPINA3K (SA3K) treatment (20 µg/eye/day). The edema and opacity of cornea were seen in (**B**) and (**C**) and were attenuated in (**D**). (**E**) The statistic analysis of inflammatory index between the groups (data are presented as Mean±SEM, n = 6 in each group). The inflammatory index was significantly reduced in the SERPINA3K-treated group on days 5 and 8, compared with the PBS or BSA-treated groups. (** p<0.01; *** p<0.001) (**F–I**) Images of H&E staining on day 8 after burn. (**F**) Control group, without alkali burn (**G**) alkali burn with PBS treatment; (**H**) alkali burn with BSA treatment; (**I**) alkali burn with SERPINA3K treatment (20 µg/eye/day). The increased thickness of cornea, the increased inflammatory cells in the stroma and the attachment of exudation to endothelium were seen in (**G**) and (**H**), and the inflammation was ameliorated in (**I**). Scale bar: 100 µm.

The alkali burn induced apparent inflammation in the cornea, i.e. edema and opacity of cornea (Fig. 2 A–D), on day 8 after alkali burn. At the same time point, the cornea with SERPINA3K treatment appeared more transparent with less edema (Fig. 2D), compared with that in the PBS or BSA-treated groups (Fig. 2B, 2C). The measurement of inflammatory index demonstrated that the SERPINA3K-treated group had significantly reduced inflammation on days 5 and 8, compared with the PBS or BSA-treated groups at the same time points (Fig. 2E, data are presented as Mean±SEM, n = 6 in each group, ** p<0.01; *** p<0.001).

Meanwhile, histological analysis of the corneal sections with H&E staining showed that SERPINA3K decreased inflammatory cell infiltration in the corneal stroma (Fig. 2G–I). The SERPINA3K treatment also attenuated the alkali burn-induced thickening of the cornea, suggesting improved corneal edema (Fig. 2G–I). The exudation attached to endothelium was also apparently decreased in the SERPINA3K treated group, compared to the PBS or BSA-treated groups (Fig. 2G–I).

### Effects of SERPINA3K on the epithelium recovery

**Figure 3:**
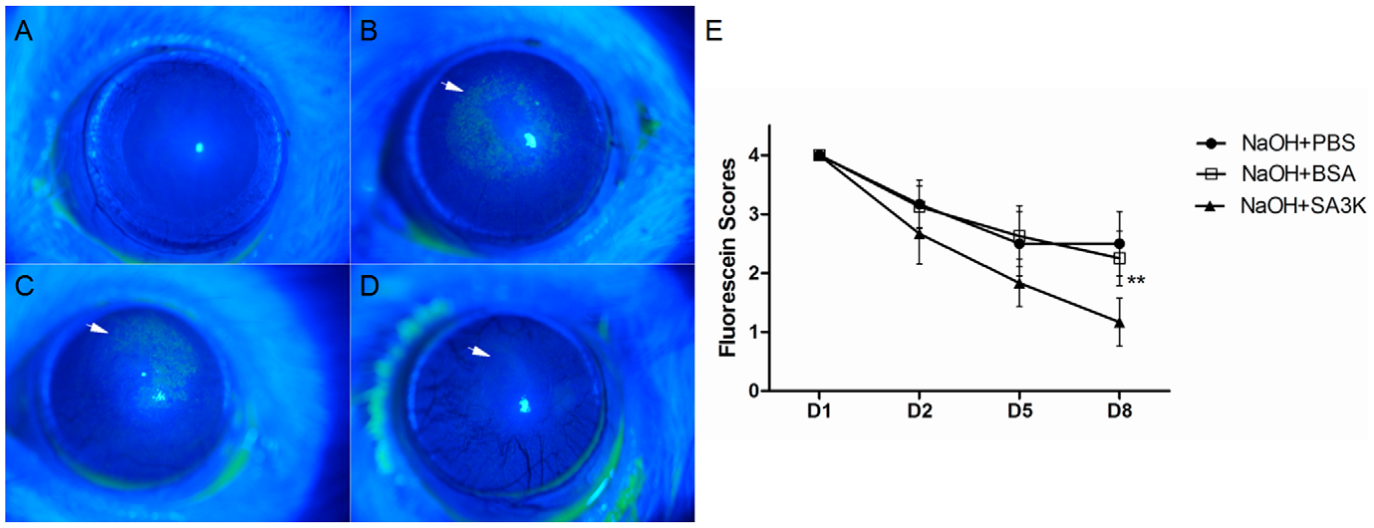
Effects of SERPINA3K on the epithelium recovery. (**A–D**) Representative images of fluorescein sodium staining of corneal on day 8 after the alkali burn. (**A**) control group, without alkali burn (**B**) alkali burn with PBS treatment; (**C**) alkali burn with BSA treatment; (**D**) alkali burn with SERPINA3K (SA3K) treatment (20 µg/eye/day). Stained dots were seen in the central cornea area of (**B**) and (**C**), the stained dots were significantly reduced in (**D**), as arrows indicate. (**E**) The statistic analysis of measurement of epithelium damage between the groups (data are presented as Mean±SEM, n = 6 in each group). The epithelium damage was significantly reduced in the SERPINA3K-treated group on day 8, compared with the PBS or BSA-treated groups. (** p<0.01).

It was demonstrated by fluorescein sodium staining that the corneal epithelium defect after alkali burn was smaller and the corneal epithelium surface became smoother in the SERPINA3K-treated group on day 8 following the alkali burn, compared to the PBS and BSA-treated groups (Fig. 3A–D). Quantification of epithelium defect showed a significant reduction of epithelium damage in the SERPINA3K-treated group, compared with the PBS or BSA-treated groups on day 8 after alkali burn (Fig. 3E, data are presented as Mean±SEM, n = 6 in each group, ** p<0.01 ).

### Down-regulation of angiogenic and inflammatory factors by SERPINA3K

**Figure 4:**
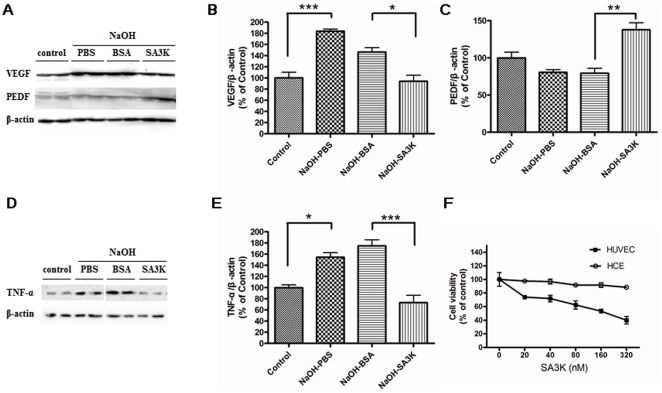
Down-regulation of anti-angiogenic and anti-inflammatory factors by SERPINA3K. (**A**) Western blotting results of VEGF and PEDF on day 8 after alkali burn. Individual lanes from left to right, 1 & 2: control group, without alkali burn; 3 & 4: alkali burn with PBS treatment; 5 & 6: alkali burn with BSA treatment; 7–8: alkali burn with SERPINA3K (SA3K) treatment (20 µg/eye/day). (**B**) The statistic analysis of Western blotting results of VEGF between the groups on day 8 (Data are presented as Mean±SEM, n = 3 in each group). The level of VEGF was significantly reduced in the SERPINA3K-treated group, compared to the BSA-treated group. (**C**) The statistic analysis of Western blotting results of PEDF between the groups (data are presented as Mean±SEM, n = 3 in each group) on day 8. The level of PEDF was significantly up-regulated in the SERPINA3K-treated group, compared to the BSA-treated group. (**D**) The Western blotting results of TNF-α on day 8 after alkali burn. The order of individual lanes was as same as (**A**). (**E**) The statistic analysis of Western blotting results of TNF-α between the groups on day 8 (data are presented as Mean±SEM, n = 3 in each group). The level of TNF-α was significantly reduced in the SERPINA3K-treated group, compared to the BSA-treated group. (**F**) The effects of SERPINA3K on the cell viability of HCE cells and HUVEC by the MTT assay (data are presented as Mean±SEM, n = 5 in each group). It showed the inhibitory effect of SERPINA3K on the cell viability of HUVEC, but not HCE cells at the concentrations given. (* p<0.05; ** p<0.01; *** p<0.001).

To investigate the mechanism underlying the anti-angiogenic and anti-inflammatory effects of SERPINA3K in the cornea, we measured pro-angiogenic and pro-inflammatory factors: VEGF and TNF-α, and endogenous anti-angiogenic factor PEDF in the cornea. As shown by Western blot analysis, alkali burn significantly induced expression of VEGF and TNF-α on day 8 after alkali burn, compared to control corneas (* p<0.05; *** p<0.001). SERPINA3K significantly down-regulated the levels of VEGF (Fig. 4A, 4B) and TNF-α (Fig. 4D, 4E) in the corneas with alkali burn, compared to the BSA-treated group (* p<0.05; *** p<0.001). In the same corneas, SERPINA3K up-regulated the level of PEDF (Fig. 4A, 4C), compared to the BSA-treated group (data are presented as Mean±SEM, n = 3 in each group, ** p<0.01). These results suggest that SERPINA3K restored the VEGF∶PEDF balance (Fig. 4A–C), which may be responsible for the beneficial effect on NV.

To investigate the differential effects of SERPINA3K on the endothelial cells and epithelial cells, HUVEC and HCE cells were employed. As shown in Figure 4F, at the concentrations of 0, 20, 40, 80, 160 and 320 nM, SERPINA3K inhibited the cell growth of HUVEC in a concentration-dependent manner, but did not significantly affect the cell viability of HCE cells, suggesting that SERPINA3K has specific inhibitory effect on endothelial cells (data are presented as Mean±SEM, n = 5 in each group).

### SERPINA3K regulates EGFR

**Figure 5:**
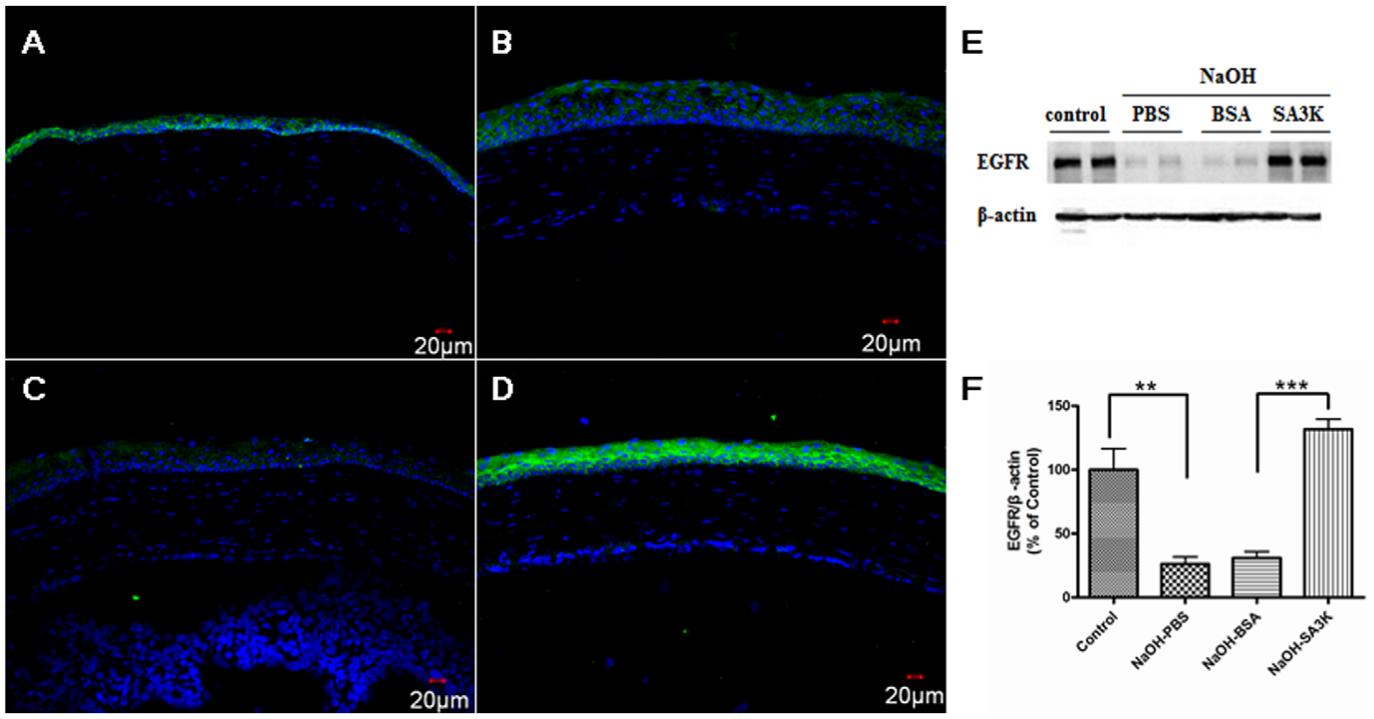
SERPINA3K regulates expression of EGFR. (**A–D**) Representative images of the immunofluorecent staining on day 8 after burn. (**A**) control group, without alkali burn (**B**) alkali burn with PBS treatment; (**C**) alkali burn with BSA treatment; (**D**) alkali burn with SERPINA3K (SA3K) treatment (20 µg/eye/day). It was shown that the expression of EGFR in the layer of epithelium cells of SERPINA3K (SA3K) treated group was apparently increased on day 8 (**D**), compared to the PBS and BSA-treated groups (**B**, **C**). (**E**) Western blotting results of EGFR on day 8 after alkali burn. Individual lanes from left to right, 1 & 2: control group, without alkali burn; 3 & 4: alkali burn with PBS treatment; 5 & 6: alkali burn with BSA treatment; 7 & 8: alkali burn with SERPINA3K treatment (20 µg/eye/day). (**F**) The statistic analysis of Western blotting results of EGFR between the groups on day 8 after alkali burn (data are presented as Mean±SEM, n = 3 in each group). The level of EGFR was significantly up-regulated in the SERPINA3K-treated group, compared to that in the BSA-treated group (** p<0.01; *** p<0.001).

To investigate the mechanism responsible for the enhanced epithelium recovery by SERPINA3K, the expression of EGFR was analyzed. As shown by the immunofluorescent staining, the expression of EGFR in the layer of epithelium cells was apparently increased in the SERPINA3K-treated group (Fig. 5D) on day 8 after the alkali burn, compared with the PBS and BSA-treated groups (Fig. 5B, 5C). Likewise, Western blot analysis showed that levels of EGFR were reduced by alkali burn but elevated by SERPINA3K significantly, compared to the PBS and BSA-treated groups (Fig. 5E, 5F, data are presented as Mean±SEM, n = 3 in each group, ** p<0.01; *** p<0.001), suggesting that the SERPINA3K-enhanced epithelium recovery after alkali burn may be through up-regulating EGFR.

## Discussion

It has been reported that SERPINA3K has anti-angiogenic, anti-inflammatory and anti-oxidant effects in the retina [20]–[22]. The application of SERPINA3K in the cornea has not been reported previously. In this present study, we provided the first evidence that SERPINA3K ameliorates corneal NV and inflammation in a corneal injury model. This study also indicated that these beneficial effects may be mediated through down-regulation of pro-angiogenic and pro-inflammatory factors and up-regulation of endogenous angiogenic inhibitors. Furthermore, our results showed that SERPINA3K also enhanced corneal epithelium recovery after the injury, possibly via up-regulating EGFR expression, a novel activity of SERPINA3K.

It has been established that VEGF is a key pro-angiogenic and pro-inflammatory factor. It plays an important role in angiogenesis and inflammation. Multiple studies suggest that blocking VEGF expression or neutralizing its activities is an effective strategy in the treatment of neovascular disorders [27], [28]. Alkali burn significantly up-regulated VEGF, which may contribute to corneal NV and inflammation in this model. The over-expression of VEGF induced by alkali burn was attenuated by SERPINA3K, which may be responsible, at least in part, for the anti-NV effect of SERPINA3K in the model. It has been reported that SERPINA3K is an inhibitor of the Wnt pathway [23]. Since the Wnt pathway is known to up-regulate angiogenic factors including VEGF, it is likely that SERPINA3K may down-regulate VEGF via blocking the Wnt pathway.

PEDF is a potent endogenous inhibitor of angiogenesis and inflammation [8], [9]. It contunter-balances the activity of VEGF and plays an important role in angiogenesis control. The disturbed balance between VEGF and PEDF has been implicated in NV. Here we showed for the first time that SERPINA3K up-regulates endogenous PEDF expression, which may be another mechanism for its anti-inflammatory and anti-angiogenic effects.

SERPINA3K and PEDF are both members of the SERPIN family, and share significant sequence homology [18]. In this study, the increased VEGF level and the decreased level of PEDF was observed after the cornea was chemically injured with alkali. The treatment of SERPINA3K significantly attenuated the VEGF over-expression induced by the alkali burn while significantly elevated the PEDF levels. These results suggest that SERPINA3K mediates angiogenesis and inflammation likely via regulating the VEGF∶PEDF balance, i.e. down-regulating VEGF and up-regulating PEDF expressioin.

Vascular endothelial cells are widely used to study the mechanism for NV [34], [35]. It has been well documented that primary HUVEC can be activated by VEGF and thus is employed as an *in vitro* model for anti-VEGF therapy or other anti-NV strategies [34], [35]. In the present study, we used HUVEC to compare the specific inhibition of SERPINA3K on the vascular endothelial cells. We demonstrated that SERPINA3K has specific inhibitory effects on the cell growth of HUVEC, but not HCE cells. Since we have found recently that SERPINA3K is an antagonist of the canonical Wnt pathway which regulates multiple angiogenic factors including VEGF and TNF-α [23]. This may represent a mechanism responsible for its inhibitory effect on endothelial cell growth. However, the mechanism by which SERPINA3K regulates EGFR remains to be studied in the future.

Tumor necrosis factor-α (TNF-α) is a commonly accepted key inflammatory factor [29]–[31]. The level of TNF-α was increased after alkali burn, which contribute to corneal inflammation. SERPINA3K attenuated the increase of TNF-α level induced by alkali burn, correlating with other beneficial effects on the pathologic changes caused by the burn, e.g. the decrease of inflammation index (Fig. 2E) and decrease of inflammatory cells in the stroma (Fig. 2I), suggesting that SERPINA3K's inhibitory effect on the inflammation of injured cornea can be at least partially ascribed to the down-regulation of TNF-α.

In contrast to its inhibitory effect on NV, our study for the first time showed that SERPINA3K enhances the recovery of epithelium after injury. EGFR is the cell-surface membrane receptor of the EGF and plays a key role in the epithelial cell proliferation and survival [32], [33]. Both our immunofluorescent staining and Western blotting analyses showed that SERPINA3K up-regulated the expression of EGFR in the cornea. The up-regulation of the expression of EGFR by SERPINA3K under pathological conditions may represent a mechanism responsible for the enhancing effect of SERPINA3K on epithelium recovery.

Corneal wounding is a common eye disease worldwide, particularly in the developing countries. Corneal wounding involves a very complicated battery of pathologic alterations, including NV, inflammation and epithelium recovery etc. The present study proved again that multiple factors, such as VEGF, PEDF, TNF-α are involved in the corneal wounding. We provided novel evidence of the new direction and hint for the treatment of corneal wounding, and SERPINA3K can become a potential therapeutic agent for the corneal wounding.
